# Basal lamina changes in neurodegenerative disorders

**DOI:** 10.1186/s13024-021-00502-y

**Published:** 2021-12-07

**Authors:** Benjamin Nguyen, Gregory Bix, Yao Yao

**Affiliations:** 1 Department of Pharmaceutical and Biomedical Sciences, University of Georgia, Athens, GA USA; 2 Clinical Neuroscience Research Center, Tulane University School of Medicine, New Orleans, Louisiana USA; 3 Departments of Neurosurgery and Neurology, Tulane University School of Medicine, New Orleans, Louisiana USA; 4 Department of Molecular Pharmacology and Physiology, Morsani College of Medicine, University of South Florida, MDC 8, Tampa, Florida, 33612 USA

**Keywords:** Basal lamina, Neurodegenerative disorders, Blood-Brain Barrier, Laminin, Perlecan

## Abstract

**Background:**

Neurodegenerative disorders are a group of age-associated diseases characterized by progressive degeneration of the structure and function of the CNS. Two key pathological features of these disorders are blood-brain barrier (BBB) breakdown and protein aggregation.

**Main body:**

The BBB is composed of various cell types and a non-cellular component---the basal lamina (BL). Although how different cells affect the BBB is well studied, the roles of the BL in BBB maintenance and function remain largely unknown. In addition, located in the perivascular space, the BL is also speculated to regulate protein clearance via the meningeal lymphatic/glymphatic system. Recent studies from our laboratory and others have shown that the BL actively regulates BBB integrity and meningeal lymphatic/glymphatic function in both physiological and pathological conditions, suggesting that it may play an important role in the pathogenesis and/or progression of neurodegenerative disorders. In this review, we focus on changes of the BL and its major components during aging and in neurodegenerative disorders, including Alzheimer’s disease (AD), Parkinson’s disease (PD), and amyotrophic lateral sclerosis (ALS). First, we introduce the vascular and lymphatic systems in the CNS. Next, we discuss the BL and its major components under homeostatic conditions, and summarize their changes during aging and in AD, PD, and ALS in both rodents and humans. The functional significance of these alterations and potential therapeutic targets are also reviewed. Finally, key challenges in the field and future directions are discussed.

**Conclusions:**

Understanding BL changes and the functional significance of these changes in neurodegenerative disorders will fill the gap of knowledge in the field. Our goal is to provide a clear and concise review of the complex relationship between the BL and neurodegenerative disorders to stimulate new hypotheses and further research in this field.

## Background

Neurodegenerative disorders, a group of age-associated diseases, are characterized by progressive neuronal dysfunction and cognitive decline. Three major types of neurological disorders are Alzheimer’s disease (AD), Parkinson’s disease (PD), and amyotrophic lateral sclerosis (ALS). Neurodegenerative disorders in total affect more than 7 million people in the US [1]. This number is expected to increase dramatically over the next few decades with our aging population. Unfortunately, there are no effective treatments or disease-modifying therapeutics for these devastating diseases.

One key pathology of neurodegenerative disorders is blood-brain barrier (BBB) breakdown, which has been observed in almost all neurodegenerative disorders in both rodents and humans [2–4]. BBB breakdown in AD has been confirmed in multiple independent postmortem human studies [5]. Recent neuroimaging studies have detected BBB breakdown in individuals with mild cognitive impairment (MCI) and early AD, before cognitive decline and/or other brain pathologies [6–15]. MRI studies also show increased cerebral microbleeds (reflecting loss of cerebrovascular integrity) in 25% of individuals with MCI and 45–78% of individuals with early AD before dementia [16–21]. These findings indicate that BBB disruption is not only a consequence but also a cause in AD [4, 7, 9, 22–24].

Vascular cognitive impairment (VCI), cognitive deficits associated with cerebrovascular disease, is another common type of dementia [25]. Like in AD, BBB disruption correlates well with VCI. For instance, serum-derived proteins are detected in brain tissue from VCI patients [26, 27]. Increased albumin and laminin levels are detected in the CSF from VCI patients [27–29]. MRI studies also demonstrate enhanced BBB leakage in VCI patients [30–33]. These results suggest an essential role of BBB breakdown in the pathogenesis of VCI.

BBB disruption has also been reported in animal models of PD [34–36]. A postmortem study revealed increased BBB permeability in the post commissural putamen of PD patients [37]. A positron emission tomography (PET) study showed dysfunction of the BBB transporter system in PD patients [38], and a dynamic contrast enhanced MRI study demonstrated enhanced BBB leakage in PD patients [39]. These results highlight a critical role of BBB integrity in PD.

Similarly, blood-CNS barrier (BCNSB) disruption has also been reported in ALS. For example, blood-spinal cord barrier (BSCB) breakdown was detected in rodent models of ALS prior to motor neuron degeneration and neuroinflammation and worsens with disease progression [40–45], although one human study reported that BSCB leakage was independent of motor neuron pathology in ALS [46]. Postmortem studies revealed structural and functional impairment of the BSCB in gray and white matter microvessels of medulla and spinal cord tissue from ALS patients [45, 47, 48]. Recent neuroimaging studies have demonstrated early BSCB dysfunction in ALS patients [49–51]. These findings suggest that barrier disruption contributes to ALS pathogenesis.

It should be noted that, although BBB breakdown is detected in other neurodegenerative diseases such as frontotemporal dementia (FTD) and dementia with Lewy bodies (DLB) [52, 53], little data are available on the role of BBB breakdown in the pathogenesis of these disorders [54, 55].

Together, these findings highlight the important role of BBB in neurodegenerative disorders. Most BBB research focuses on its cellular components, including brain microvascular endothelial cells (BMECs), pericytes, and astrocytes [4, 56]. For example, pericytes have been shown to play a key role in BBB maintenance [57–59] and loss of pericytes contributes to BBB disruption and pathogenesis of various neurodegenerative diseases [50, 60–62]. Its non-cellular component---the basal lamina (BL), on the other hand, is largely understudied. Recent studies from our laboratory and others show that the BL actively regulates BBB integrity and abnormal BL is observed in various neurological disorders [63–65].

Another common pathology of neurodegenerative disorders is protein aggregation. For example, accumulation of β-amyloid (Aβ)/tau, alpha-synuclein, and TDP-43/SOD-1 is a hallmark of AD, PD, and ALS, respectively [66–70]. Although how exactly these aggregates form is not fully understood, reduced clearance is an important mechanism. Recent evidence suggests that receptor-mediated transport across the BBB and the meningeal lymphatic/glymphatic system are two major mechanisms of solute/waste clearance in the brain, and that both mechanisms are affected in aging and neurodegenerative diseases [71–87]. Since the BL is a key constituent of the BBB and meningeal lymphatic/glymphatic system, we hypothesize that BL defects contribute to the development of neurodegenerative disorders via compromising BBB integrity and/or affecting protein aggregate clearance.

## Main body

### CNS blood vessels

**Fig. 1:**
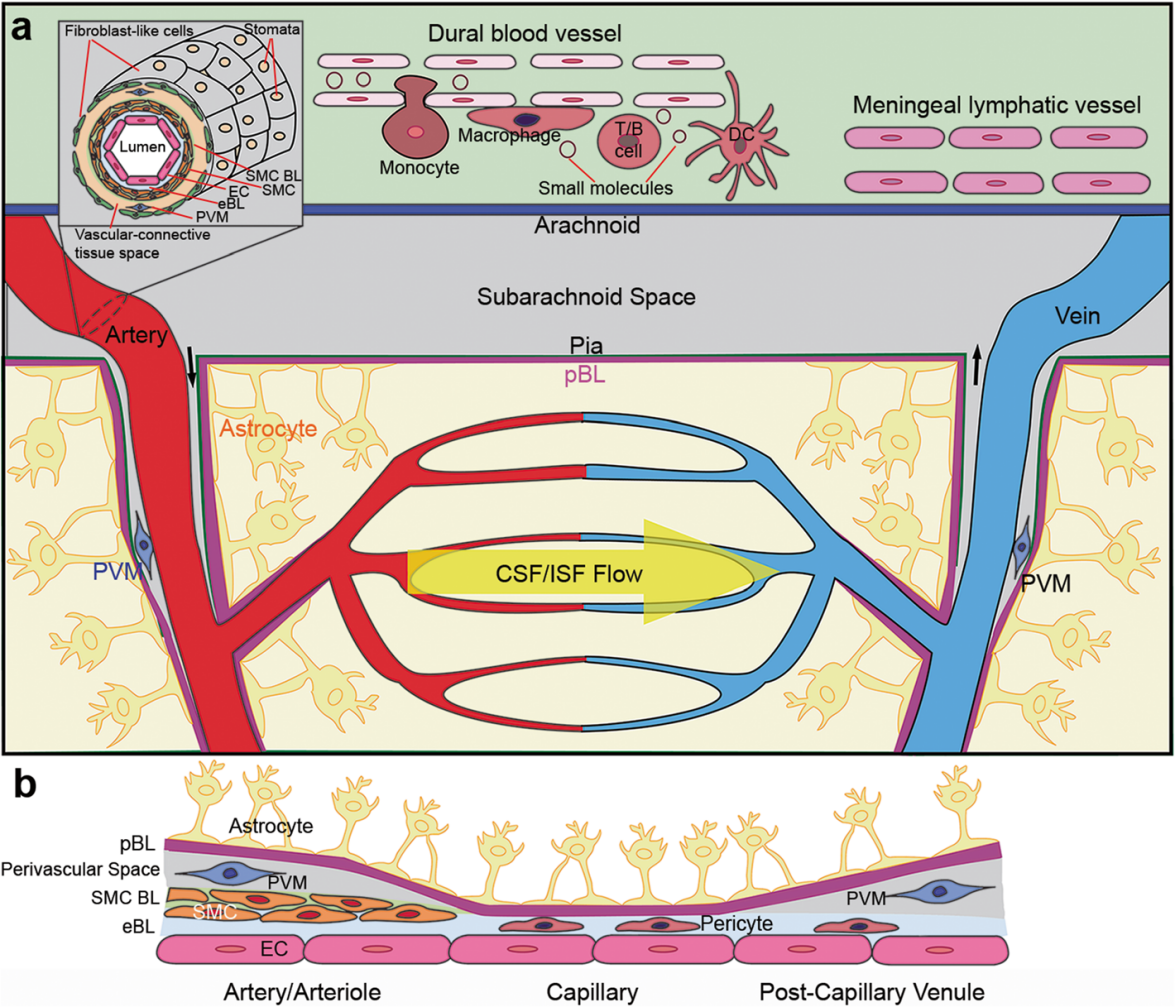
Diagram illustration of the vasculature and BL layers in the CNS. **a** Scheme of the CNS blood vessels and meningeal lymphatic/glymphatic system. The inset highlights the cross-section view of a meningeal artery. **b** Scheme of various BL layers and cellular components at the arterial/arteriole, capillary, and post-capillary venule levels in the CNS vasculature. BL: basal lamina; eBL: endothelial basal lamina; EC: endothelial cell; pBL: parenchymal basal lamina; PVM: perivascular macrophage; SMC: smooth muscle cell

In the CNS, blood vessels exist in both the meninges (dura matter and leptomeninges) and brain/spinal cord parenchyma. Meningeal blood vessels exhibit unique features depending on the location. Specifically, dural blood vessels are fenestrated and lack tight junctions (Fig. 1a), enabling the entry of large molecules and immune cells from the blood into the dura matter [88–90]. Echoed with this finding, a large repertoire of immune cells, including T cells, B cells, dendritic cells, macrophages, and mast cells, are found in the dura matter in steady-state conditions [89, 91–99]. Leptomeningeal blood vessels are non-fenestrated and have tight junctions (Fig. 1a). Although lacking astrocytic ensheathment, these leptomeningeal vessels are impermeable to large molecules [88, 89, 100]. Similarly, immune cells also exist in the leptomeninges, but to a lesser extent compared to dura matter [89, 91–99]. In addition, leptomeningeal vessels are covered by stomata-containing adventitia lining cells (fibroblast-like cells) (Fig. 1a), which may be involved in cerebral spinal fluid (CSF)/interstitial fluid (ISF) exchange [101]. It remains unclear how exactly meningeal blood vessels change during aging and in neurodegenerative disorders. This is an interesting field for future research.

Large arteries and veins in the leptomeninges penetrate brain/spinal cord parenchyma and branch into arterioles and venules, which are connected via capillaries (Fig. 1a). The composition of vascular wall changes along the arterial-venous axis, although endothelial cells and astrocytic endfeet are found in all sections of the vasculature. Specifically, smooth muscle cells, perivascular macrophages, and multiple (endothelial, smooth muscle cell [SMC], and parenchymal) BL layers are found in arteries/arterioles (Fig. 1b). Pericytes and tightly packed endothelial and parenchymal BLs exist in capillaries (Fig. 1b). Similar structure is observed in post-capillary venules, with endothelial and parenchymal BLs separated by a perivascular space (Fig. 1b). Accumulating evidence suggests that the structure and function of parenchymal blood vessels are altered during aging and in neurodegenerative disorders. For example, pericyte loss and abnormal BL are observed in AD brains in both rodents and humans [9, 59, 60, 102–106].

### CNS lymphatic system

In the brain, lymphatic vessels are located in dural meninges along sinuses (Fig. 1a) [88, 107–110]. The presence of “leaky” blood vessels and lymphatic vessels enables immune cells to migrate to and from the dura matter, making the meninges an immunologically active barrier tissue [89]. It has been suggested that meningeal lymphatics function to sample and clear CSF/ISF into the cervical lymph nodes [107–115].

Unlike the meninges, CNS parenchyma lacks lymphatic vessels. How is waste removed from the CNS? Using mice with intra-cisterna magna injection of fluorescent tracers, Iliff and colleagues proposed a para-vascular route, known as the glymphatic system [71, 116]. The glymphatic system posits that CSF in the subarachnoid space circulates into brain parenchyma along para-arterial spaces via connective flow. It crosses the glia limitans and enters the brain extracellular spaces in an aquaporin-4 (AQP4)-dependent manner. The CSF then mixes with ISF and moves toward the para-venous spaces. Metabolic waste eventually exits the CNS via deep veins, meningeal/cervical lymphatic vessels, and perineural sheaths of cranial/spinal nerves. Consistent with this hypothesis, reduced CSF influx was observed in four different AQP4^-/-^ mouse lines and Snta1^-/-^ mice, which lack perivascular AQP4 [71, 117]. In addition, the clearance of SOD1 oligomer was significantly delayed in AQP4^-/-^ mice compared to the controls [118].

It should be noted, however, that there is also evidence that does not support the proposed glymphatic mechanism or convective pressure-driven CSF flow from para-arterial to para-venous spaces through parenchymal extracellular spaces. For example, in contrast to previous reports [71, 117], one study showed that loss of AQP4 failed to affect solute transport from the subarachnoid space to brain parenchyma in rodents [119]. In addition, recent animal and modeling studies have concluded that fluid flow in brain extracellular spaces is predominantly diffusive rather than convective in nature [119–125]. Considering all these findings, an updated glymphatic system with convective flow along perivascular spaces of large vessels and diffusion in brain extracellular spaces has been proposed (Fig. 1a) [73, 101, 117, 126, 127]. Given that the BL is a major constituent of the perivascular space [102, 128–130] and many BL components regulate AQP4 expression [131–134], it is speculated that the BL may regulate protein clearance via meningeal lymphatic/glymphatic system in neurodegenerative disorders.

Recent studies have shown that meningeal immunity (immune cells in the dura matter) is affected during aging and under neurodegenerative conditions [91–95]. The exact role of each immune population, however, remains largely unknown and needs future research. In addition, the CSF/ISF drainage function of the meningeal lymphatic/glymphatic system is substantially impaired in aging. It has been reported that aged mice exhibit decreased para-vascular recirculation of CSF, diminished CSF/ISF exchange, and loss of perivascular AQP4 polarization [76, 107]. Echoed with this report, reduced meningeal lymphatic function is associated with compromised macromolecule drainage and cognitive decline in old mice [107, 114, 135]. Interestingly, VEGF-C, a lymphatic endothelial cell mitogen, is able to increase meningeal lymphatic drainage and improve cognitive function in old mice [135]. Based on these results, it is hypothesized that impaired meningeal lymphatic drainage attenuates the clearance of abnormal protein aggregates and thus aggravates disease pathology/outcome. Consistent with the hypothesis, disruption of meningeal lymphatic vessels in young 5xFAD mice promotes Aβ deposition in the meninges and exacerbates parenchymal Aβ burden [135]. Similarly, significantly reduced meningeal lymphatic flow and delayed deep cervical lymph node perfusion were found in PD patients [136]. Ablation of meningeal lymphatic drainage resulted in meningeal inflammation, enhanced PD pathology, and exacerbated motor/memory deficits in mice injected with α-synuclein preformed fibrils [136]. Echoed with this finding, impaired meningeal drainage caused α-synuclein accumulation, glial activation, increased inflammatory cytokines, and neuronal loss in α-synuclein_A53T_ PD mice [137]. In addition, a 35% decrease in the clearance of intra-cisterna magna-injected ovalbumin was observed in SOD1_G93A_ mouse model of ALS [118]. Together, these results suggest an essential role of the CNS lymphatic system in the pathogenesis of neurodegenerative disorders.

### Basal lamina

**Fig. 2:**
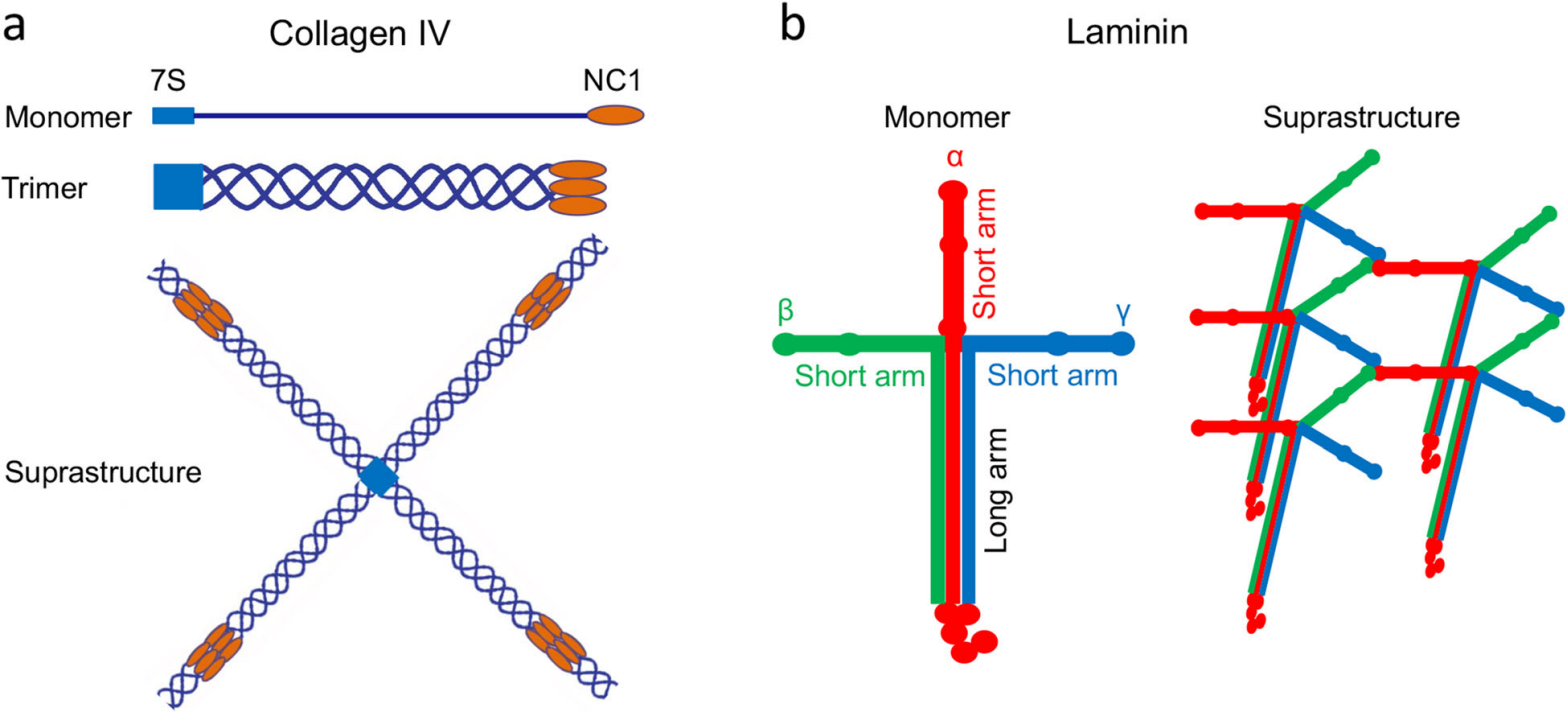
Structural illustration of major BL components collagen and laminin. **a** Collagen IV monomer, trimer, and its suprastructure. 7S, 7S domain; NC1, NC1 domain. **b** Laminin monomer and its suprastructure. α, β, and γ: laminin-α, -β, and -γ chains, respectively

The BL is a complex amorphous structure with a thickness of 50-100nm. It lays beneath epithelial and endothelial cells, and is a major constituent of the BBB and CNS lymphatic system [128, 138–141]. In the CNS, multiple BL layers, including endothelial BL, SMC BL, and parenchymal BL, are found between BMECs and astrocytic endfeet [142–144] (Fig. 1b). Biochemically, the BL contains four major extracellular matrix (ECM) proteins: collagen IV, laminin, nidogen, and heparan sulfate proteoglycans (HSPGs). Minor BL constituents include fibulins, osteonectin, and netrin-4 [145]. These components are predominantly synthesized by BMECs, pericytes, and astrocytes in the brain. It should be noted that the BL is also found in peripheral blood vessels. Although peripheral BL contains all four major ECM proteins, it lacks astrocyte-derived ECM proteins compared to CNS BL. In this section, we discuss the structure (Fig. 2) and function (Table 1) of major components of the BL in physiological conditions.

**Table 1:** Loss-of-function studies on major BL components

Targets	Mutations	Cre lines	Phenotypes	References
Collagen 4A1/2	Global KO	-	Embryonic lethality (E10.5-11.5), BM structural deficiencies	[64]
	Missense mutations	-	Vascular defects, brain damage of differing severity	[146–148]
Collagen 4A1	Loss of exon 41 in both alleles	-	Embryonic lethality, ICH	[146]
	Loss of exon 41 in one allele	-	Perinatal lethality with ICH, Porencephaly	[149, 150]
	Conditional knockout	Tie2-Cre	ICH, Increased retinal vascular branching, Porencephaly, Macroangiopathy	[151]
		PDGFRβ-Cre	ICH, Increased retinal vascular branching, Porencephaly, Macroangiopathy	[151]
		GFAP-Cre	Very mild ICH, No defects in retinal branching	[151]
Laminin α2	Global knockout	-	BBB disruption	[132, 152]
Laminin α4	Global knockout	-	Disrupted vascular integrity, Hemorrhage at perinatal stage	[153]
Laminin α5	Global knockout	-	Embryonic lethality (~E17)	[154–156]
	Conditional knockout	Tie2-Cre	No gross abnormalities	[157–159]
Laminin β1	Global knockout	-	Embryonic lethality (E5.5-6.5)	[160]
Laminin γ1	Global knockout	-	Embryonic lethality (E5.5-6.5)	[160–162]
	Conditional knockout	Nestin-Cre	BBB breakdown, ICH	[132, 163]
		CamK2a-Cre	No BBB breakdown or ICH	[132, 163]
		PDGFRβ-Cre	BBB breakdown and hydrocephalus in C57Bl6/FVB mixed background	[134]
			Age-dependent mild BBB breakdown without hydrocephalus in C57Bl6 dominant background	[133]
		SM22α-Cre	No gross abnormalities	[134, 164]
Nidogen 1	Global knockout	-	Grossly normal, Upregulation of nidogen 2	[165–169]
Nidogen 2	Global knockout	-	Grossly normal	[170]
Nidogen 1/2	Global knockout	-	Perinatal lethality, BM defects	[65, 171, 172]
Agrin	Global knockout	-	Embryonic lethality	[173]
	Conditional knockout	Tie2-Cre	Intact BBB structure, Reduced AQP4 expression	[131]
Perlecan	Global knockout	-	Embryonic lethality (E10-12), Developmental defects, BM deterioration in areas with high mechanical stress	[174–176]
	Hypomorph (C1532Yneo)	-	Reduced perlecan secretion, skeletal phenotype similar to Schwartz-Jampel syndrome	[177]
	C1532Y		Normal perlecan secretion, grossly normal	[177]
	Knockout rescued	-	Viable and intact BBB under homeostatic conditions, exacerbated BBB damage and attenuated pericyte accumulation after ischemic stroke	[178]

#### Collagen IV

Collagen IV is the most abundant component in the BL and is mainly produced by endothelial cells, astrocytes, and pericytes [179–183]. Structurally, collagen IV is a trimeric protein containing three α-chains (Fig. 2a). Currently, six different collagen IV α-chains have been identified (COL4A1-6) [184–186]. By dimerization at its NC1 domain and tetramerization at its 7S domain, collagen IV forms a sheet-like suprastructure [187, 188] (Fig. 2a).

Loss-of-function studies reveal critical roles of collagen IV in embryonic development and vascular integrity. Genetic knockout of COL4A1/2 results in embryonic lethality at E10.5 - E11.5 due to BL structural deficiencies, although BL formation at earlier stages is unaffected [64]. Similarly, loss of COL4A1 exon 41 in both alleles causes embryonic lethality and intracerebral hemorrhage (ICH) [146], while its deficiency in one allele leads to perinatal lethality with ICH (~50%) and porencephaly in some survivors [149, 150]. Furthermore, the function of COL4A1 has also been investigated in a cell-specific manner. Deletion of COL4A1 in BMECs or pericytes results in ICH, increased retinal vascular branching, porencephaly, and macroangiopathy [151]. Ablation of COL4A1 in astrocytes, however, causes very mild ICH without defects in retinal vascular branching [151]. Consistent with these findings, missense mutations in COL4A1 and COL4A2 lead to vascular defects and/or brain damage to different degrees [146–148]. Collectively, these studies suggest that collagen IV is nonessential for initial BL assembly but plays a key role in BL maintenance and vascular integrity [56].

#### Laminin

Laminin is a trimeric protein composed of α, β, and γ chains [63, 106, 128] (Fig. 2b). These subunits bind to one another via their C-terminal domains, forming a T- or cross-shaped molecule composed of one long arm and two or three short arms (Fig. 2b). Like collagen IV, laminin is able to form a sheet-like suprastructure by intermolecular self-polymerization via their N-terminal domains [189, 190] (Fig. 2b). Currently, 5 α, 4 β, and 3 γ chains have been identified [128]. Various combinations of these subunit variants create different laminin isoforms. However, it should be noted that not all combinations have been confirmed. Among the 60 theoretical isoforms, only 16 have been identified and 4 proposed based on *in vitro* and *in vivo* studies so far [128]. Since distinct laminin isoforms may have different functions, it is important to study laminin’s function in an isoform-specific manner.

In the brain, laminin is predominantly synthesized by BMECs, pericytes, and astrocytes [56]. Interestingly, these cells synthesize and deposit different laminin isoforms to the BL. Specifically, BMECs mainly generate laminin-α4β1γ1 (-411) and laminin-511 [142, 191], and astrocytes predominantly produce laminin-211 [142, 192]. Immunocytochemical analysis from our laboratory shows that brain pericytes primarily generate α4/α5- and γ1-containing laminins [134, 181], although laminin-α2 is also detected in pericytes by single-cell RNAseq analysis [193]. Therefore, laminin-411 and -511 (derived from BMECs) are predominantly found in the endothelial BL, while laminin-211 (derived from astrocytes) is mainly found in the parenchymal BL.

Many loss-of-function studies have been performed to investigate laminin’s function in BBB integrity. Such studies heavily rely on conditional knockout mice, since most global knockouts (e.g. laminin-α5^-/-^, -β1^-/-^, and -γ1^-/-^) are embryonic lethal [154–156, 160–162], hindering further investigation into their roles in BBB integrity.

Global knockout of laminin-α4 leads to disrupted vascular integrity and hemorrhage at perinatal stage, but not in adulthood [153]. The lack of phenotype in adulthood is believed to be compensated by laminin-511, which is expressed in blood vessels at postnatal stage [153, 194]. To investigate the functional significance of laminin-α5, we and others generated endothelial (Tie2-Cre) laminin-α5 conditional knockout mice. Interestingly, these mutants fail to show a phenotype under homeostatic conditions [157–159], suggesting that laminin-411 may be able to compensate for the loss of endothelial laminin-511. To overcome this mutual compensation between laminin-411 and -511 and investigate the role of endothelial laminin, we further generated mice lacking both laminin-α4 and -α5 in endothelial cells. The resulting mutants display moderate BBB breakdown, characterized by enhanced leakage of intravenously injected tracers into brain parenchyma. These findings strongly suggest that endothelial laminins maintain BBB integrity.

To investigate the function of astrocytic laminin (laminin-211) in BBB integrity, we generated conditional knockout mice with laminin-γ1 deficiency in neural progenitor cells (Nestin-Cre) and in neurons (CamK2a-Cre), respectively [132]. The former but not the latter show severe BBB disruption and spontaneous ICH [132, 163], highlighting an important role of glial cell-derived laminin in BBB regulation. Using adenovirus expressing Cre under GFAP promoter, we further validated that loss of astrocytic laminin leads to BBB breakdown [132]. Consistent with our result, mice with global knockout of laminin-α2 demonstrate BBB disruption [132, 152]. Together, these findings suggest an indispensable role of astrocytic laminin in BBB maintenance.

Due to the lack of pericyte-specific markers [62], the function of pericytic laminin in BBB integrity was investigated indirectly using two conditional knockout mouse lines. By using the PDGFRβ-Cre line, we generated mice with laminin deficiency in mural cells, which include both pericytes and SMCs [134]. By using the SM22α-Cre line, we generated mice with laminin deficiency in SMCs only [134, 164]. Although the SMC-specific laminin knockout mice fail to show gross abnormalities [134], the mural cell-specific laminin knockout mice display genetic background-dependent phenotypes. In C57/Bl6-FVB mixed background, the mutants exhibit BBB breakdown and hydrocephalus with incomplete penetrance [134]. In C57Bl6 dominant background, these mutants are grossly normal at young age, but develop mild BBB compromise at old age [133]. Together, these findings suggest that pericyte- rather than SMC-derived laminin also contributes to BBB maintenance, but to a lesser extent compared to astrocytic or endothelial laminin.

#### Nidogen

Nidogen, also called entactin, is a glycoprotein containing three globular and multiple rod-like domains [195]. Unlike collagen IV and laminin, nidogen is unable to self-polymerize or form a sheet-like suprastructure. Instead, it interacts with both collagen IV and laminin as a crosslinker. It has been suggested that nidogen may play a role in stabilizing collagen IV and laminin networks.

Two nidogen isoforms (nidogen-1/2) have been identified in mammals [165]. Global knockout of either isoform results in a generally normal phenotype [165–168, 170], indicating possibly mutual compensation. Interestingly, there is a redistribution and upregulation of nidogen-2 in nidogen-1 knockout mice, although nidogen-1 expression is unaffected in nidogen-2 knockout mice [169, 170]. Consistent with the mutual compensation hypothesis, loss of both isoforms simultaneously leads to early perinatal death and severe BL defects [65, 171, 172]. These results suggest that these two isoforms share a compensatory mechanism, and nidogen function in BBB integrity remains unknown due to early perinatal lethality. Nidogen-1/2 double conditional knockout mice may overcome embryonic lethality and enable investigation of its role in BBB maintenance. Future research should focus on generating this mutant line.

#### HSPG

HSPGs are glycoproteins containing covalently attached heparan sulfate (HS) chains. They are present on cell surface of all tissues and in the BL. Their functions vary depending on the types of HSPGs. Two major HSPGs found in the BL are agrin and perlecan.

##### Agrin

Agrin is a multidomain HSPG [196–198], which can interact with laminin [199]. There are two isoforms of agrin (z+ and z-), but only the z- isoform is present in the BL [196, 200, 201]. It has been shown that agrin accumulates in the BL during BBB development in a correlation study [202], suggesting a possible role of agrin in early BBB development. Global knockout of agrin results in embryonic lethality [173], preventing investigation of its function at later stages. Interestingly, mice with agrin deletion in endothelial cells (Tie2-Cre) display intact BBB structure, although AQP4 expression is reduced [131], suggesting that agrin is dispensable for the structural but not biochemical maintenance of the BBB. The exact function of agrin in BBB integrity needs further investigation.

##### Perlecan

Perlecan, also called HSPG2, is a large HSPG with five domains and three glycosaminoglycan chains [128, 203]. Like nidogen, perlecan is unable to form a sheet-like suprastructure [203], but can interact with other BL components and/or heparin-binding growth factors [203–206]. Perlecan global knockout embryos exhibit severe developmental defects in several organs, including the brain, and usually die around E10 - E12 [174–176]. Interestingly, BL formation is not affected in these mutants, although BL deterioration is observed in areas with high mechanical stress [175]. This data indicates that perlecan is not required for BL assembly but plays an important role in BL maintenance. To overcome embryonic lethality, several perlecan mutant mouse lines have been generated. For example, mice carrying a C-to-Y mutation at residue 1532 with the neomycin cassette (C1532Yneo) exhibit reduced perlecan secretion and a skeletal phenotype similar to Schwartz-Jampel syndrome, whereas those harboring only the mutation without the neomycin cassette (C1532Y) display comparable perlecan level and no obvious abnormalities [177]. It should be noted that whether the C1532Yneo mice have BBB disruption remains unclear. Recently, a perlecan-knockout rescued line was generated by expressing perlecan gene under the Col2α1 promoter and enhancer in the perlecan^-/-^ background [178]. These mutants are viable and show no defects in BBB integrity [178], indicating a dispensable role of perlecan in BBB maintenance under homeostatic conditions. After ischemic stroke, however, these mutants demonstrate exacerbated BBB damage and attenuated pericyte accumulation [178]. Mechanistic studies reveal that perlecan promotes PDGFRβ signaling and pericyte migration [178]. These results suggest that perlecan contributes to BBB repair in ischemic stroke possibly by regulating pericyte recruitment.

### Basal lamina and normal aging

**Fig. 3:**
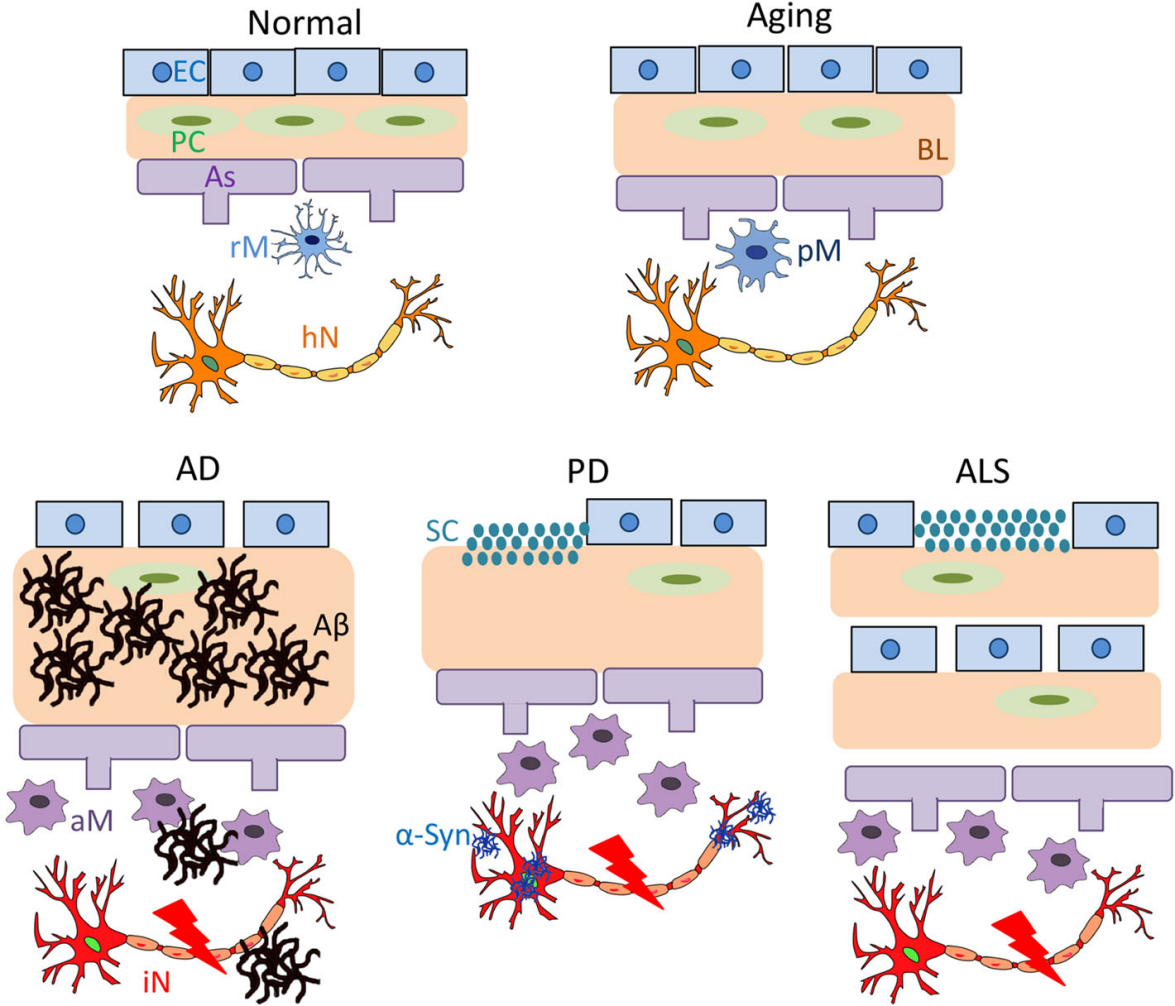
BL changes in aging and neurological disorders. Diagram illustrations showing changes of BL, endothelial cells, pericytes, microglia, neurons, and protein aggregates in aging and three neurodegenerative disorders (AD, PD, and ALS). BL: basal lamina; EC: endothelial cell; PC: pericytes; As: astrocytes; rM: resting microglia; pM: primed microglia; aM: activated microglia; hN: healthy neurons; iN: injured neurons; Aβ: β-amyloid; α-Syn: α-synuclein; SC: serum components; AD: Alzheimer’s disease; PD: Parkinson’s disease; ALS: amyotrophic lateral sclerosis

BL defects are observed during normal aging. The functional significance of these BL alterations, however, is largely unknown. In this section, we summarize (Table 2) and illustrate (Fig. 3) how the BL and its major components change during normal aging.

**Table 2:** Changes of the BL and its major components during normal aging

Normal Aging		Changes	References
**BL**	**Rodents**	Thickening	[207–209]
	**Humans**	Thickening	[210–212]
**Collagen IV**	**Rodents**	↑	[209]
		↓	[207]
		↔	[66]
	**Humans**	↑	[210, 211]
		↔	[212]
**Laminin**	**Rodents**	↑	[209]
		↓	[66, 207]
	**Humans**	↓	[211]
**Nidogen**	**Rodents**	↑	[209]
		↓	[207]
	**Humans**	↑	[212]
**Agrin**	**Rodents**	Unknown	
	**Humans**	↑	[211]
**Perlecan**	**Rodents**	↑	[66, 207, 209]
	**Humans**	Unknown

#### The BL is thickened during aging

Accumulating evidence shows that the BL thickens with age in rodents, although the extent of BL thickening is much less during normal aging compared to neurodegenerative disorders [207–209]. Consistent with these findings, mild BL thickening is also observed in aged human brains [210–212]. Together with slightly increased BL, mild pericyte loss and microglial priming are also observed in aged brains [59, 62, 213, 214]. These results suggest an important role of the BL in aging and age-associated alteration of BL composition.

#### Collagen IV alteration during aging is controversial

Controversy exists on how collagen IV changes during normal aging. On one hand, increased collagen IV levels have been found in the CNS in both rodents and humans [209–211]. In sharp contrast to these reports, one study found decreased collagen IV in aged mouse brain [207]. In addition, unaltered collagen IV levels have also been reported during normal aging in both mice and humans [66, 212]. This discrepancy may be caused by different experimental methods and conditions. Future research should address how exactly collagen IV changes during normal aging.

#### Laminin expression is decreased during aging

Most studies show that laminin level decreases during normal aging. For example, reduced laminin expression has been reported in aged mice [66, 207] and humans [211]. One study, however, finds increased laminin expression in mouse retina during aging [209]. This disparity may be explained by different laminin antibodies and/or fixation protocols used in these studies. It has been shown that heavy formaldehyde fixation masks laminin antigen, while mild-moderate fixation reveals it [215].

#### Nidogen alteration during aging is controversial

During normal aging, both decreased [207] and increased [209] nidogen levels have been reported in mouse brains. In postmortem human brains, however, increased nidogen expression is observed during normal aging [212]. It is important to determine how nidogen changes during normal aging in future research.

#### Agrin alteration during aging is largely unknown

Few studies investigated how normal aging affects agrin levels. One group found increased agrin in human brains during normal aging [211]. This finding needs to be validated in future studies.

#### Perlecan expression is elevated during aging

Mounting evidence supports that perlecan expression increases during normal aging [66, 207, 209]. It should be noted, however, that these studies were all performed in mice. It is critical to validate this finding in human brains in the future.

### Basal lamina and Alzheimer’s disease

AD, first reported in 1907, is the most common form of dementia. Clinically, AD is characterized by brain shrinkage and progressive decline in memory and other cognitive functions, which usually lead to behavioral changes, impaired mobility, hallucinations, seizures, and ultimately death [216, 217]. Pathologically, AD is marked by senile plaques (SPs), neurofibrillary tangles (NFTs), and cerebral amyloid angiopathy (CAA). SPs are extracellular aggregates composed of Aβ peptides, while NFTs are intracellular accumulation of hyperphosphorylated tau protein [216]. CAA, found in ~80% of AD cases, refers to the deposition of Aβ in cerebral and leptomeningeal blood vessel wall [218]. Depending on if there is a genetic cause, AD is categorized into familial AD (fAD) and sporadic AD (sAD). The former is caused by mutations of certain genes, including amyloid precursor protein (APP) and presenilin-1/2 (PSEN1/2) [216]. The latter occurs in a sporadic nature without known causes. The APOE gene is considered to be the biggest risk factor for sAD, with APOE4 heterozygotes and homozygotes having 3x and 12x higher risks, respectively [219].

The pathogenesis of AD remains largely unknown, although many theories have been proposed. One of the most popular hypotheses is the amyloid cascade hypothesis (ACH), which has been the foundation of numerous studies and potential therapies [220]. It proposes that genetic and environmental factors cause deposition of Aβ and formation of SPs and NFTs, eventually leading to neuronal loss and dementia [221]. This hypothesis is supported by the findings that Aβ is the most important constituent of SPs [222] and abnormal processing of APP is an early event of AD [221, 223]. It, however, has been challenged by the following observations. First, a significant amount of elderly people present Aβ plaques without developing any clinical symptoms of AD [224], suggesting that other factors may also contribute to AD pathogenesis. Second, fAD patients have abnormal Aβ production at young age but do not develop AD symptoms until at old age [225]. Third, the presence of SPs/NFTs is quantitatively inconsistent with cognitive impairment, age, and disease progression [226–228]. Lastly, no ACH-based therapies have been successfully developed [229], although this could be due to a number of other reasons.

Recently, an alternative “two-hit” hypothesis has been proposed. This hypothesis highlights the importance of vascular factors, in addition to genetic and environmental factors [5, 22]. It proposes that vascular damage causes BBB dysfunction and hypoperfusion, which compromise the clearance of Aβ and other neurotoxins, leading to neurodegeneration and dementia [22, 230]. The vascular and neurodegenerative pathways can act independently or synergistically [5, 22]. This hypothesis is supported by several findings. First, BBB disruption and hypoperfusion occur prior to Aβ accumulation and dementia in both APP_swe/E693G_ mice and AD patients [231]. Next, BMECs and pericytes are activated/degenerated in the early symptomatic stage of AD, which impairs Aβ clearance and blood flow [4, 10, 60, 61], leading to accumulation of metabolic waste and toxins, microglial activation, and eventually neuronal death.

Understanding how exactly BBB integrity and function are regulated in AD will shed light on the pathogenesis of AD and may identify novel targets with therapeutic potential. Given that the BL is actively involved in BBB maintenance [63, 102, 106, 128, 129, 218] and Aβ clearance [71, 77, 88, 102, 130]. it is logical to speculate that BL defects may contribute to AD pathogenesis. Here, we summarize (Table 3) and illustrate (Fig. 3) how the BL and its major constituents change in AD, and discuss their functional significance.

**Table 3:** Changes of the BL and its major components in AD

AD		Changes	Models	References
**BL**	**Rodents**	Thickening	3xTG, PSEN1_P117L,_ APP_swe/E693G_	[231–234]
	**Humans**	Thickening	-	[210, 212, 235–242]
		Thinning	-	[243]
**Collagen IV**	**Rodents**	↑	3xTG	[232, 234]
		↓	APP_swe_, APOE4	[244, 245]
		↔	PSEN1_P117L_	[233]
	**Humans**	↑	-	[210, 235, 237–239, 241, 246, 247]
		↔	-	[212]
**Laminin**	**Rodents**	↑	APP_swe/E693G_	[231]
		↓	APOE4	[244]
		↔	PSEN1_P117L_	[233]
	**Humans**	↑	-	[248, 249]
**Nidogen**	**Rodents**	Unknown		
	**Humans**	↓	-	[212]
**Agrin**	**Rodents**	↔	APOE4	[244]
	**Humans**	↑	-	[235, 250]
		↓	-	[243]
**Perlecan**	**Rodents**	↔	APOE4	[244]
	**Humans**	↑	-	[235]

#### The BL is thickened in AD

Mounting evidence suggests that the BL thickens in AD brains in both transgenic mouse models (regardless of transgenic genes) and postmortem human samples [210, 212, 231–242], although one study finds BL thinning in postmortem human brains [243]. Interestingly, BL thickening in AD brains displays a region-specific pattern: it is mainly observed in the cerebral cortex, hippocampus, and thalamus [207, 237]. Furthermore, an immuno-electron microscopy study shows that BL thickening predominantly occurs in parenchymal BL rather than endothelial BL [237]. These findings establish a direct association of BL thickening with AD.

Based on that BL thickening occurs before Aβ deposition in the vessel wall [231], it is hypothesized that BL thickening compromises Aβ clearance. Two major pathways are responsible for Aβ clearance in the CNS: receptor-mediated transport across the BBB and meningeal lymphatic/glymphatic system. It has been shown that 85% of Aβ is cleared via transport across the BBB and 15% via ISF bulk flow under physiological conditions [80–87]. In AD brains, however, the receptor-mediated trans-vascular mechanism is severely compromised. One major receptor that mediates Aβ uptake and transport across the BBB is low-density receptor-related protein 1 (LRP1) [80, 84, 251]. Both animal [81, 252, 253] and human [80, 81, 254] studies demonstrate that LRP1 expression at the BBB is significantly reduced in aging and AD. Additionally, inactivation of endothelial LRP1 activates the cyclophilin A-matrix metalloproteinase 9 pathway in endothelial cells, which reduces collagen IV and tight junction proteins, resulting in BBB impairment and cognitive deficits [255]. Similarly, activation of the same pathway in pericytes causes BBB breakdown in mice deficient in murine ApoE or those expressing human ApoE4, which poorly interacts with LRP1 [104].

In addition, Aβ clearance via the meningeal lymphatic/glymphatic system is also impaired in AD. First, APOE4 transgenic mice show severely disrupted perivascular drainage of Aβ and brain accumulation of Aβ [244]. Next, decreased CSF influx and Aβ clearance rate are observed in both aged AD mice with existing Aβ plaques and young AD mice without visible Aβ plaques [66, 256]. Third, reduced glymphatic flow is found in WT mice with intraventricular or intra-hippocampal injection of Aβ [207, 256], suggesting that Aβ itself can disrupt glymphatic flow. Like in AD mice, AD patients show reduced CSF uptake and attenuated clearance of tau tracer F-THK5117, which are associated with increased Aβ levels in the brain [109, 257]. Together, these results suggest that BL thickening is an early event, which contributes to AD pathogenesis by impairing Aβ clearance.

Tauopathy is another key feature of AD. Accumulating evidence suggests that tau pathology is also correlated with BBB dysfunction [258]. For example, truncated tau has been shown to increase endothelial permeability via activating glial cells in an *in vitro* BBB model, although it is not directly toxic to endothelial cells [259]. Tau-induced glial activation has been shown to enhance the expression of endothelial adhesion molecules and increase the transmigration of leukocytes across the BBB [260]. In addition, mice expressing human tau protein in astrocytes exhibit BBB disruption in areas with robust astrocytic tau pathology [261]. Progressive IgG leakage and T cell infiltration are also observed in brain tissue from the tetracycline-regulatable rTg4510 tau transgenic mice [262]. Interestingly, this BBB impairment correlates with the appearance of perivascular tau around major hippocampal blood vessels and is reversible when tau expression was suppressed [262]. Furthermore, BBB breakdown has also been reported in tauopathies without Aβ pathology, including progressive supranuclear palsy [262]. Similarly, BBB disruption is detected in regions of dense perivascular p-Tau accumulation in chronic traumatic encephalopathy [263]. These findings suggest that tau alone can compromise BBB integrity. It remains unclear, however, that how BL thickening affects tau pathology in AD. This question needs to be answered in future studies.

What causes BL thickening in AD brains? One possibility is that imbalance of different BL components leads to BL thickening [264]. Echoed with this hypothesis, it has been shown that APOE4 is able to bind BL components, including laminin and HSPGs [265, 266], and affect BL composition [244]. In addition, all BL constituents are able to interact with Aβ and affect its biochemical properties by inhibiting, disassembling, or strengthening Aβ fibrillation. Here, we discuss the changes and functions of four major components of the BL in AD individually.

#### Collagen IV alteration in AD is controversial

Collagen IV shows inconsistent alterations in transgenic AD mouse models. For example, increased collagen IV expression is found in the BL of 3xTG (APP_swe_/PSEN1_M146V_/Tau_P301L_) transgenic mice [232, 234], decreased collagen IV level is observed in APP_swe_ (APP_K670N, M671L_) [245] and APOE4 [244] transgenic mice, while unaltered collagen IV level is reported in PSEN1_P117L_ mice [233]. Unlike mouse studies, the vast majority of postmortem human studies reveal increased collagen IV levels in AD brains [210, 235, 237–239, 241, 246, 247], although one study finds unchanged collagen IV in AD brains [212]. Interestingly, the upregulation of collagen IV in AD brains is region-specific. Collagen IV is mainly increased in the frontal and temporal cortex in both subclinical (Braak stage 3-4) and AD (Braak stage 5-6) patients [235, 241], while it is not severely altered in the neocerebellum [267]. The discrepancy on collagen IV changes in AD may be explained by different genetic models and tissues (mouse vs. human). It should be noted that each genetic model has its own limitations and that no animal models can fully replicate AD pathology in patients.

The functional significance of collagen IV changes in AD is unknown. *In vitro* studies show that collagen IV is able to bind APP with high affinity [268, 269], prevent the formation of β-sheet structured Aβ40 aggregates [270], and induce disassembly of Aβ42 fibrils [271]. These results suggest that enhanced expression of collagen IV is a protective mechanism, and that increasing collagen IV expression may have a therapeutic potential in AD. The exact function of collagen IV in AD, however, needs to be investigated *in vivo* using both loss-of-function and gain-of-function studies in the future. Collagen IV conditional knockouts or hypomorphs may be useful in loss-of-function studies, while mice overexpressing collagen IV or recombinant collagen IV may be useful in gain-of-function studies.

#### Laminin alteration in AD is controversial

Similarly, controversial results exist on how laminin changes in AD. One report finds decreased laminin expression in the BL in APOE4 mouse brains [244], while other studies show unaltered [233] or increased [231, 248, 249] laminin expression in AD brains. One possible reason for this discrepancy is that pan-laminin rather than subunit-specific antibodies were used in these studies. Pan-laminin antibodies, such as the polyclonal antibody against Engelbreth Holm-Swarm mouse sarcoma-derived laminin, are unable to distinguish different laminin subunits in the BL, and thus cannot reflect changes of individual laminin isoforms. Given that distinct laminin isoforms exert different functions [63, 106, 128, 129], it is important to determine how each individual laminin isoform changes. It has been reported that laminin-α1 and -γ1 levels are substantially increased in human AD brains, primarily in reactive astrocytes and/or SPs [248, 249]. Future studies should determine how other laminin subunits change in AD using subunit-specific antibodies.

Although the function of laminin in AD remains largely unclear, there is evidence suggesting that laminin may negatively regulate AD pathogenesis by promoting Aβ clearance. First, it has been reported that laminin is able to inhibit the formation of Aβ40 fibrils [272–274] and disrupt fibrils that have already formed *in vitro* [275]. Next, laminin at high concentration induces a random structural transition of Aβ42 fibrils, disassembling the β-sheet structure and inhibiting fibrillation [271]. Consistent with these findings, laminin is able to interact with amyloid precursor protein [276] and co-localizes with Aβ or Aβ-APOE4 complex in AD brains [250, 277]. In addition, it has been proposed that APOE regulates Aβ clearance in an isoform-specific manner, possibly due to distinct affinity for laminin [84, 277]. With the generation of various laminin conditional knockout mouse lines, the function of each laminin isoform in AD pathogenesis can be investigated *in vivo* in future studies. Currently, there are no known disease-modifying therapies for AD. Laminin may be a promising target, given its ability to inhibit the formation of Aβ40/Aβ42 fibrils and promote Aβ clearance. Further studies, however, are needed to investigate the therapeutic potential of laminin in AD.

#### Nidogen alteration in AD is largely unknown

How nidogen changes in AD is mostly unknown. There is only one such study, which finds decreased nidogen expression in CAA in postmortem human brains [212]. This knowledge gap should be addressed in future research.

Our understanding of nidogen’s role in AD predominantly comes from *in vitro* studies. It has been shown that nidogen dose-dependently inhibits Aβ40 fibril formation and induces disassembly of Aβ40 and/or Aβ42 fibrils *in vitro* [271], suggesting a beneficial role of nidogen in AD. Elucidating nidogen’s function in AD *in vivo* relies on loss-of-function studies. There are two nidogen isoforms in mammals [165]. Genetic ablation of either isoform results in a normal phenotype [165–168, 170], suggesting functional compensation between these isoforms. Knockout of both isoforms simultaneously, however, leads to perinatal lethality [128], preventing investigation of nidogen’s role in AD *in vivo*. Although both single knockout mice fail to show gross abnormalities under homeostatic conditions, they may display exacerbated vascular and/or neurological damage in AD background. In addition, conditional knockout of both nidogen isoforms may overcome perinatal lethality and enable investigation of nidogen’s function in AD pathogenesis. Future research should focus on generating these mutant mice.

#### HSPG in general is increased in AD

It has been reported that HSPG in general has a 9.3-fold increase in the hippocampus and 6.6-fold increase in the superior frontal gyrus in AD patients [278].

Unlike other major BL constituents, which inhibit Aβ fibrillation, HSPGs have been found to accelerate Aβ fibrillation contributing to AD pathogenesis [279–281]. First, HSPGs are able to bind Aβ and affect its fibrillation via their HS chains [282]. It has been shown that the sulfation pattern of HS is associated with its affinity for Aβ [283] and Aβ fibrillation capability [284]. For example, sulfated HS has a higher affinity for Aβ, while desulfated HS loses Aβ-binding capability almost entirely [283]. Removal of heparin’s O-sulfate partially inhibits Aβ aggregation, while deletion of all sulfate groups completely abolishes Aβ fibrillation [284]. Next, HSPGs also inhibit Aβ degradation [285]. *In vitro* studies have demonstrated that HSPGs inhibit the proteolytic degradation of fibrillar Aβ [286], and Aβ prevents proteolytic breakdown of HSPGs by inhibiting heparanase, the enzyme that degrades HS [287]. These findings suggest that Aβ-HS interaction protects each other from degradation [288]. Consistent with this observation, overexpression of heparanase lowers Aβ burden in APP transgenic mice [289]. Subsequent mechanistic study reveals that heparanase exerts this function by either releasing Aβ from the plaques or inhibiting tau fibril formation and propagation [288]. Together, these results suggest that HSPGs contribute to almost every stage of AD pathogenesis, including Aβ aggregation and clearance, and that decreasing HSPGs or increasing heparanase activity may have a therapeutic potential in AD.

##### Agrin alteration in AD is controversial

There are controversial findings on how agrin changes in AD brains. Specifically, while unaltered agrin expression is observed in APOE4 mice [244], both decreased [243] and increased [235, 250] agrin levels have been reported in AD patients. Biochemical analysis shows that all agrin from normal brains is soluble in 1% SDS, whereas a large fraction of agrin from AD brains is insoluble [290]. Since fibrillar Aβ exhibits similar solubility properties [290], it is suggested that agrin may interact with Aβ. This is consistent with the observation that agrin is detected in SPs, NFTs, and CAA in AD brains [250, 279, 280, 291, 292]. Future studies should determine how exactly agrin changes in AD brains in both mice and humans.

In contrast to HSPGs, agrin has been shown to inhibit Aβ deposition in the brain. For example, overexpression of agrin leads to decreased Aβ [131]. Consistent with this finding, deletion of endothelial agrin in APPswe/PSEN1_dE9_ mice results in increased Aβ40 deposition, although loss of neuron-associated agrin in these mutants fails to affect Aβ [131]. In addition, agrin may be also involved in Aβ clearance via the glymphatic system. It has been shown that ablation of endothelial agrin in APPswe/PSEN1_dE9_ mice reduces AQP4 expression in astrocytic endfeet [131], and that deletion of AQP4 in AD mice results in Aβ accumulation without affecting the expression of Aβ-degrading proteases [293]. These results are consistent with previous reports that the function of glymphatic system is AQP4-dependent [294–296]. Together, these findings suggest that agrin negatively regulates AD pathogenesis by inhibiting Aβ deposition and promoting Aβ clearance, and that upregulating agrin expression may be able to improve AD outcome.

##### Perlecan alteration in AD is controversial

Perlecan has been shown to increase in human AD brains [235], but unaltered in APOE4 mice [244]. It remains unclear whether perlecan is a key component of SPs, NFTs, and CAA in AD brains. Several studies fail to detect perlecan in SPs, NFTs, and CAA in AD brains [291, 292, 297], while a group reports its presence in these structures [298]. This discrepancy may be caused by different perlecan antibodies and/or experimental approaches. How perlecan changes in mouse and human AD brains should be clarified using various antibodies and standard protocols.

Like agrin, perlecan also shows a neuroprotective role against Aβ toxicity. It has been reported that perlecan domain V inhibits neurotoxic signaling cascade by blocking Aβ-integrin-α2β1 interaction *in vitro* [299, 300]. In addition, perlecan domain V is able to reverse Aβ toxicity in endothelial cells and restore angiogenesis *in vitro* [301]. These results suggest a therapeutic potential of perlecan in AD. It should be noted, however, that it remains unclear how perlecan regulates Aβ deposition/clearance and AD pathology *in vivo*. This gap of knowledge can be addressed by using perlecan hypomorphic (C1532Yneo) mice and/or perlecan-knockout rescued mutants in future studies.

### Basal lamina and Parkinson’s disease

PD, first medically described by James Parkinson in 1817, is the second most common neurodegenerative disease after AD [302]. Typical symptoms of PD include resting tremor, gait disorders, and bradykinesia [303]. In addition, various nonmotor symptoms, such as olfactory dysfunction, depression/anxiety, and dementia, are also found in PD patients [303, 304]. Although there is no cure for PD, some symptomatic treatments have been found beneficial. Among all PD cases, approximately 95% occur sporadically without known genetic causes, while 5% can be attributed to gene mutations [305, 306]. These genes include SNCA (α-synuclein), LRRK2 (leucine rich repeat kinase 2), VPS35 (vacuolar protein sorting 35 homolog), and GBA (glucocerebrosidase) [304, 307, 308].

Pathologically, PD is characterized by loss of dopaminergic neurons in the brain, most dramatically in the pars compacta of the substantia nigra and locus coeruleus [309]. A hallmark of PD is the formation of Lewy bodies, which contain aggregated and post-translationally modified α-synuclein. Various studies report lower α-synuclein level in the CSF of PD patients [310–313], suggesting that defective transport/clearance of α-synuclein may contribute to PD pathogenesis. This is consistent with previous findings that α-synuclein can be transported bidirectionally across the BBB [310], and that α-synuclein is overexpressed in melanized neurons in PD patients [314]. There is evidence showing that BBB integrity is compromised in PD brains [37, 315–317]. Given the important functions of BL in BBB maintenance and the meningeal lymphatic/glymphatic system, it is reasonable to speculate that BL defects may contribute to PD pathogenesis. Here, we summarize (Table 4) and illustrate (Fig. 3) how the BL and its major components change in PD, and discuss their functional significance.

**Table 4:** Changes of the BL and its major components in PD

PD		Changes	Models	References
**BL**	**Rodents**	Unknown		
	**Humans**	Thickening	-	[241, 318]
		Collapsed	-	[319]
**Collagen IV**	**Rodents**	↑	α-synuclein_A30P_	[320]
	**Humans**	↑	-	[241, 318]
**Laminin**	**Rodents**	Unknown		
	**Humans**			
**Nidogen**	**Rodents**	Unknown		
	**Humans**			
**Agrin**	**Rodents**	Unknown		
	**Humans**			
**Perlecan**	**Rodents**	Unknown		
	**Humans**			

#### The BL is thickened and collapsed in PD

Although there are no reports on BL changes in PD mouse models, BL abnormalities have been observed in PD patients. Specifically, capillary BL thickening [241, 318] and collapsed BL without endothelium [319] are found in postmortem brains of PD patients. The degeneration of endothelial cells exposes the BL to serum proteins, which leads to abnormal accumulation of plasma proteins at the BL, degeneration of pericytes, activation of glial cells, and eventually neuronal death [319]. Future studies should elucidate how BL changes in various PD mouse models.

The functional significance of BL changes in PD remains unknown. On one hand, BL thickening may be a protective mechanism to attenuate PD pathology. For example, it is possible that the BL thickens to enhance α-synuclein clearance via the meningeal lymphatic/glymphatic system. On the other hand, BL thickening may be the consequence of α-synuclein accumulation in the vasculature. In this case, BL thickening has a deleterious role in PD. Future studies should distinguish these possibilities and explore the molecular mechanisms underlying BL thickening in PD. Here, we discuss the changes and functions of four major components of the BL in PD individually.

#### Collagen IV is increased in PD

Most studies find increased collagen IV level in PD brains. For example, enhanced expression of COL4A2 has been observed in the brains of α-synuclein_A30P_ mice [320]. Echoed with this finding, collagen IV accumulation is also found in postmortem brains from PD patients [241, 318]. Although one study finds no alterations in total length and density of collagen IV^+^ blood vessels in PD patients, collagen IV intensity is not reported in this study [319]. Since many other genes are associated with fPD, it is important to determine if collagen IV displays similar changes in other PD mouse models in the future.

Although the functional significance of collagen IV upregulation in PD is unknown, there are several hypotheses. One possibility is that enhanced collagen IV may contribute to PD pathogenesis by inducing ER stress. In support of this hypothesis, abnormal expression of collagen IV has been reported to increase ER stress [321], and a strong association of altered Golgi morphology and COL4A2 upregulation is observed in PD models [320]. Another possibility is that upregulation of collagen IV may alter BL morphology and function, and thus affect BBB integrity and brain influx/efflux function. In addition, collagen IV may also affect PD pathogenesis by regulating α-synuclein aggregation in the brain. Future studies should focus on testing these hypotheses and determining the exact role of collagen IV in PD.

#### Laminin alteration in PD is unknown

There are no studies examining how laminin alters in PD brains. Future research should fill this gap of knowledge by determining how each individual laminin isoform changes in PD brains using subunit-specific antibodies.

Accumulating evidence suggests that laminin exerts a neuroprotective role in PD. First, laminin has been shown to act as a neurite outgrowth-promoting factor for neurons *in vitro* [322]. Next, laminin-HSPG complex is able to enhance neurite outgrowth *in vitro* [323]. Similarly, peptide nanofibers with HS mimetic and laminin-derived epitopes significantly promote neurite outgrowth *in vitro* [324]. Consistent with these *in vitro* studies, the KDI domain of laminin-γ1 has been shown to protect rat dopaminergic neurons from 6-hydroxydopamine-induced toxicity [325], highlighting a beneficial role of γ1-containing laminins in PD. In addition, HS-mimetic and laminin-mimetic peptide amphiphile nanofibers substantially enhance dopamine and tyrosine hydroxylase levels, reduce cleaved Cas-3 level, and improve function and tissue integrity in the 6-hydroxydopamine-induced PD model in rats [326], indicating a therapeutic potential of laminin and HS in PD. It is unclear, however, whether this beneficial effect is from laminin, HS, or both.

In addition, it remains unknown which laminin isoforms mediate the neuroprotective function in PD. There is evidence suggesting that laminin-511 exerts a beneficial role in PD. Specifically, laminin-511 has been shown to promote the survival and differentiation of midbrain dopaminergic neurons by inducing miR-130a to suppress PTEN (phosphatase and tensin homolog) [327, 328]. Since laminin-511 is mainly produced by BMECs [142, 191], which undergo degeneration in PD [319], it is hypothesized that BMEC degeneration-induced loss of laminin-511 significantly contributes to PD. This hypothesis, however, needs further investigation.

#### Nidogen alteration in PD is unknown

There are no reports on how nidogen changes in PD brains. This is an interesting area for future research.

The function of nidogen in PD is unknown. This is predominantly due to the mutual compensation between two nidogen isoforms [165–168, 170] and embryonic lethality of the double knockouts [65, 171, 172]. It is interesting to see if nidogen single knockout mice develop any phenotype in PD background. In addition, mice with conditional knockout of both nidogen isoforms may enable investigation of nidogen’s function in PD pathogenesis in a cell-specific manner. Future research should focus on generating these mutant mice.

#### HSPG alteration in PD is unknown

Like laminin and nidogen, how HSPG changes in PD brains remains unknown. This important question needs to be answered in future studies.

The function of HSPGs in PD remains unclear. However, there are reports showing that laminin-HSPG complex exerts a beneficial role in PD. For instance, laminin-HSPG complex is able to enhance neurite outgrowth *in vitro* [323]. In addition, not only do peptide nanofibers with HS mimetic and laminin-derived epitopes induce neurite outgrowth *in vitro* [324], they also reduce brain injury and enhance functional recovery in a rat model of PD [326]. These results suggest a neuroprotective role of HS and laminin in PD. Whether this beneficial effect is from HS, laminin or both, however, remains elusive. Since HSPGs are absent in Lewy bodies and do not regulate the fibrillization & stabilization of Lewy bodies [329], it is more likely that the above-described neuroprotective function is from laminin rather than HSPGs. This possibility, however, needs further investigation.

### Basal lamina and amyotrophic lateral sclerosis

ALS, first described in 1869, is a progressive nervous system disease clinically characterized by muscular weakness and paralysis. It affects approximately 2 in every 100,000 people, and ~16,000 Americans are living with ALS at any time [330]. Unfortunately, there are no effective disease-modifying therapies at present. The typical pathology of ALS is motor neuron degeneration in the spinal cord, motor cortex, and brain stem. Like AD and PD, the vast majority of ALS cases are sporadic (sALS), while only ~10% are familial (fALS). fALS is genetically linked to at least 15 genes, including SOD1 (Cu/Zn superoxide dismutase), TARDBP (TAR DNA-binding protein), FUS (fused in sarcoma), ANG (angiogenin precursor), and OPTN (optineurin) [331]. Approximately 20% of fALS cases are caused by a missense mutation in SOD1 [332]. The mutant SOD1 forms intracellular aggregates, which alter gene expression and protein interactions, leading to motor neuron death via toxic hydroxyl radicals [333]. In addition, mounting evidence suggests that BBB integrity is disrupted in both sALS and fALS. For example, serum protein leakage, tight junction protein reduction, pericyte loss, astrocytic endfeet detachment and degeneration, and BL component changes have been reported independently in ALS in both humans [3, 45, 47, 48, 334–336] and mice [40–45]. Since BBB disruption occurs before motor neuron degeneration and neuroinflammation [42, 43, 45], it is believed that BBB breakdown actively contributes to ALS. Given the critical role of BL in BBB maintenance, we hypothesize that BL defects may contribute to ALS pathogenesis. Here, we summarize (Table 5) and illustrate (Fig. 3) how the BL and its major components change in ALS, and discuss their functional significance.

**Table 5:** Changes of the BL and its major components in ALS

ALS		Changes	Models	References
**BL**	**Rodents**	Thickening, duplication	SOD1_G93A_	[40]
	**Humans**	Exposed to plasma proteins	-	[48]
**Collagen IV**	**Rodents**	↑	SOD1_G93A_	[45]
	**Humans**	↑	-	[48]
		↓	-	[45, 337, 338]
		↔	-	[339]
**Laminin**	**Rodents**	↓	SOD1_G93A_	[41]
	**Humans**	↑	-	[340, 341]
		↔	-	[339]
		↓	-	[342]
**Nidogen**	**Rodents**	Unknown		
	**Humans**			
**Agrin**	**Rodents**	↓	SOD1_G93A_	[43, 343]
	**Humans**	↔	-	[339]
**Perlecan**	**Rodents**	Unknown		
	**Humans**			

#### The BL is thickened in ALS

Abnormalities in the BL and its surrounding structures are observed in ALS. First, BL thickening and multiple layers of BL and BMECs are detected in the spinal cord and brain stem in the SOD1_G93A_ mouse model of ALS [40]. In addition, reduced pericyte density, retracted astrocytic endfeet, and extracellular edema are also found in these mutants [40, 344]. In sALS patients, the BL is exposed to plasma proteins through detached BMECs, which leads to accumulation of fibrin and collagen IV within the BL [48]. This BL abnormality may contribute to pericyte degeneration and astrocyte defects. Future research should address if similar BL defects are observed in ALS patients.

Although a correlation between BL defects and ALS severity is observed, it remains unclear if BL changes are a cause or consequence of ALS. Based on that BL thickening and duplication occur at early symptomatic stage in SOD1_G93A_ mice [40], it is speculated that these BL changes may be a defense mechanism to BMEC degeneration. In addition, these BL defects may also contribute to ALS pathogenesis via affecting BBB integrity and possibly the meningeal lymphatic/glymphatic pathway. It has been shown that BMEC detachment exposes the BL to plasma proteins, leading to BL thickening and enhanced vascular permeability [40, 334]. These possibilities should be assessed in future studies.

#### Collagen IV alteration in ALS is controversial

Controversial results exist on how collagen IV changes in ALS. On one hand, increased collagen IV is observed in the spinal cords of SOD1_G93A_ mice at 18 weeks of age [45]. Collagen IV-expressing microglia start to appear in the anterior horn in these mice at 15 weeks of age [45]. Similarly, collagen accumulation has been found in capillary BL in the medulla and spinal cords of ALS patients [48]. On the other hand, decreased perivascular collagen IV has been reported in ALS patients [45, 337]. Additionally, reduced or unaltered collagen IV levels are observed in non-neural tissues in ALS patients. For example, it has been reported that collagen IV is decreased in the skin and serum [338], while unaffected in the muscle of ALS patients [339]. This discrepancy may be explained by different experimental protocols, species, regions, and/or cell types. Based on that collagen IV is mostly increased in glial cells but reduced in other cells in ALS [45], it is speculated that there is a compensatory upregulation of collagen IV in glial cells in response to reduced collagen IV or other components of the BL in ALS. This hypothesis, however, needs further investigation.

The significant alteration of collagen IV in neural tissue suggests an important role of collagen IV in ALS. There are at least two different interpretations. First, the enhanced expression of collagen IV could be caused by upregulation in glial cells. In this case, collagen IV may exert a neuroprotective role to alleviate ALS injury. Alternatively, the high level of collagen IV in glial cells could be caused by increased uptake. In this case, collagen IV may exert a detrimental role to aggravate ALS injury. Future studies should focus on distinguishing these two possibilities and elucidating the function of collagen IV in ALS pathogenesis.

#### Laminin alteration in ALS is controversial

Inconsistent findings exist on how laminin changes in ALS. Using a laminin-α1/β1 antibody, it has been reported that laminin level is reduced in the spinal cords of symptomatic SOD1_G93A_ mice [41]. Similarly, laminin-α2 and -β2 are decreased in muscle BL, laminin-α4 is absent in limb muscles but not extraocular muscles in ALS patients [342], while pan-laminin is unaltered in muscles in ALS patients [339]. In contrast to these findings, laminin γ1 is strongly stained in astrocytes in white matter along the cervical and thoracic spinal cords in ALS patients, which correlates with disease severity [340]. Echoed with this result, increased laminin-111 expression has been reported in the skin of ALS patients [341]. This disparity can be explained by different laminin antibodies. Since pericyte deficits [50] and BMEC/astrocyte degeneration [40] are observed in ALS, changes in pericytic, endothelial, and/or astrocytic laminins are also expected. Subunit-specific laminin antibodies should be used to elucidate how each individual laminin isoform changes in ALS in future studies.

Although the function of laminin in ALS pathogenesis remains largely unknown, its substantial alteration in ALS suggests a crucial role of laminin in this disorder. Given that laminin-γ1 is significantly upregulated in astrocytes of ALS patients [340], and that the KDI domain of laminin-γ1 is neuroprotective in both glutamate-induced excitotoxicity [345] and 6-hydroxydopamine-induced neuronal death [325], it is speculated that laminin-γ1 may play a beneficial role in ALS [340]. This hypothesis, however, needs further investigation. In addition, the functions of other laminin subunits/isoforms in ALS should also be determined in future studies.

#### Nidogen alteration in ALS is unknown

There are currently no studies examining how nidogen changes in ALS. This question should be answered in future research.

The functional significance of nidogen in ALS is unknown, mainly due to mutual compensation between nidogen-1/2 and embryonic lethality of the double knockout mice. Similarly, nidogen single knockout mice should be crossed into the ALS background to determine if loss of either isoform affects ALS pathology. In addition, mice with conditional deletion of both nidogen isoforms simultaneously may enable investigation of nidogen’s function in ALS in a cell-specific manner. Future research should focus on generating these genetic tools.

#### HSPG alteration in ALS

##### Agrin expression is reduced in ALS

Agrin has been found to substantially reduced in both spinal cords [43] and neuromuscular junctions [343] in symptomatic but not pre-symptomatic SOD1_G93A_ animals. It remains unclear how agrin changes in spinal cords from ALS patients, although no alteration is detected in neuromuscular junctions [339]. This disparity may be explained by distinct experimental methods and/or different species.

The correlation between agrin reduction and ALS symptoms in SOD1_G93A_ mice suggests that agrin may play an essential role in the onset of symptoms. Given that agrin regulates AChR clustering and other features of postsynaptic membranes in the muscle [346], it is hypothesized that agrin may modulate symptomatic onset by affecting receptor clustering and/or postsynaptic function. This hypothesis, however, needs further investigation.

##### Perlecan alteration in ALS is unknown

Currently, there are no studies examining perlecan alteration in ALS or the functional significance of these changes. These important questions need to be answered in future studies.

## Perspectives and conclusions

Thanks to advances in genetic and biochemical techniques, significant progress has been made in BL research and neurodegenerative disorders. There are, however, several key challenges that need to be addressed and several key questions that need to be answered in future research.

First, the BL is not unique to blood vessels in the CNS. Located at the abluminal side of endothelial cells, the BL covers the entire vasculature. However, due to the contribution of astrocyte-derived ECM proteins, CNS BL has different composition and possibly distinct functions compared to peripheral BL. It should be noted that most studies reporting BL alterations in neurodegenerative disorders examined BL in the CNS. Whether and how peripheral BL changes in these conditions, however, remain unknown. If peripheral BL does change in neurodegenerative disorders, *what is the timeline and how is it compared to that of other hallmarks of the diseases (e.g. BBB breakdown)?* Answers to these questions will determine if BL changes specifically influence BBB function and if BL changes are early events in the pathogenesis of neurodegenerative disorders.

Second, controversial findings exist on how BL and its major components change in neurodegenerative disorders. This is mainly caused by the use of different antibodies and detection methods. For example, many previous studies used pan-laminin antibodies, which make it impossible to determine the changes of individual laminin isoforms. To address this issue, subunit-specific antibodies and standard protocols should be used in future studies.

Next, the BL is difficult to study due to its unique features. As a high organized structure composed of ECM proteins, the BL exhibits low solubility, high crosslinking, and heavy glycosylation [56, 106, 129, 347–349]. The BL is more resistant to detergent-based dissolving/extraction methods compared to other proteins. It is thus challenging to accurately determine the composition of the BL. Innovative techniques that enable separation of the BL from other cellular components and subsequent unbiased assays, such as decellularization followed by mass spectrometry, would address this issue. For example, various decellularization protocols have been developed and employed to isolate the kidney, heart, and liver BL in mice [350–353]. This approach has enabled direct visualization of the BL in situ and subsequent analyses [354]. Successful decellularization of CNS tissues will allow accurate assessment of BL biochemical properties (e.g. BL composition) in various neurodegenerative disorders and substantially move the field forward.

Fourth, the functions of BL and its major components in neurodegenerative disorders are largely unknown. One major challenge is embryonic lethality of many knockout mice, which prevents loss-of-function studies in adulthood. Another challenge is functional compensation among different isoforms, which leads to a grossly normal phenotype in the single knockouts. Embryonic lethality may be overcome by using conditional knockout mice. Mutual compensation can be addressed by using compound knockout mice, in which multiple isoforms are abrogated simultaneously. Generating these tools will allow us to define the functional significance of the BL and its major components in various neurodegenerative disorders.

Fifth, the causes of many neurological disorders are still unclear. Although transgenic animals replicate many features of the diseases, it should be noted that genetic models only represent familial cases, and most cases of neurodegenerative diseases are sporadic rather than familial. In addition, since multiple genes are linked to AD, PD, or ALS, there are multiple animal models for these diseases. To ensure unbiased and strong conclusions, findings from one animal model should be validated in other animal models and postmortem human samples (both familial and sporadic).

Sixth, targeting the BL therapeutically is challenging. Although BBB impairment is observed in almost all neurodegenerative disorders, only small molecules can cross the compromised BBB in the early phase of these diseases. Given that most BL components are large ECM proteins, intact recombinant proteins are usually ineffective as therapeutics. Short fragments from these proteins, such as key signaling domains, may have a therapeutic potential. This approach requires a thorough understanding of the biochemical properties of major BL components. Alternatively, endogenous levels of BL components may be targeted. This can be achieved by modulating their synthesis and/or degradation using either small-molecular compounds or virus-based approaches. Currently, there are no known small molecules that are able to regulate the expression and/or metabolism of ECM proteins. Although there are safety concerns, virus-based approaches can regulate ECM protein levels in a cell-specific manner. This allows manipulation of certain isoforms of ECM proteins specifically, which may reduce unwanted off-target effects. These therapeutic options should be explored in future studies.

## Data Availability

Not applicable.

## Abbreviations

Aβ: β-amyloid
ACH: amyloid cascade hypothesis
AD: Alzheimer’s disease
ALS: amyotrophic lateral sclerosis
ANG: angiogenin precursor
APP: amyloid precursor protein
AQP4: aquaporin-4
BBB: blood-brain barrier
BM: basement membrane
BMECs: brain microvascular endothelial cells
CAA: cerebral amyloid angiopathy
CSF: cerebrospinal fluid
ECM: extracellular matrix
FUS: fused in sarcoma
GBA: glucocerebrosidase
HS: heparan sulfate
HSPGs: heparan sulfate proteoglycans
ICH: intracerebral hemorrhage
ISF: interstitial fluid
LRP1: low-density receptor-related protein 1
LRRK2: leucine rich repeat kinase 2
NFTs: neurofibrillary tangles
OPTN: optineurin
PD: Parkinson’s disease
PSEN1/2: presenilin-1/2
SNCA: α-synuclein
SOD1: Cu/Zn superoxide dismutase
SPs: senile plaques
TARDBP: TAR DNA-binding protein
VPS35: vacuolar protein sorting 35 homolog
SMCs: smooth muscle cells
